# An Iterative Unfolding Procedure

**DOI:** 10.6028/jres.068A.039

**Published:** 1964-08-01

**Authors:** Ronald P. Uhlig

## Abstract

An iterative procedure for unfolding the effects of the finite resolution of a detector from an observed pulse height distribution is discussed. The process is demonstrated for a particular detection system. Convergence and uniqueness properties of the method are discussed empirically.

A general expression for the propagated error resulting from errors in the detected pulse height distribution is derived. Approximations are made in order to evaluate the propagated error for a particular detector. These approximations become better as the resolution of the detector improves. The results indicate that the error rapidly approaches a limit of from 1.5 to 3 times the error in the observed distribution. This limit is reached in approximately three iterations.

## 1. Introduction

In measuring γ-ray spectra it is frequently necessary to remove the effects of the resolution of the detector from an observed pulse height distribution. This is known as “unfolding”, “unscrambling”, or “unsmearing”. To do this a matrix representing the response of the detector must be found. Let the incident spectrum be denoted by an *m* dimensional vector *N*:

$$N=(\begin{array}{c} n_{1}\\ n_{2}\\ \vdots \\ n_{m} \end{array}).$$

The response may be represented by an *m×m* matrix *R.* The detected pulse height distribution, *P*, is then given by

$$P_{i}=\sum_{j}R_{ij}N_{j}.$$ (1)

Unfolding is the name given to the process of finding *N* such that

$$N_{j}=\sum_{j}R_{ij}^{-1}P_{i},$$

where *R_if_*^−1^ is the inverse to the matrix *R_tj_*.

It is frequently undesirable to obtain a solution *N_j_* by inverting the response function matrix. Usually the response function matrix is a very large square matrix. In this experiment one form of the matrix was 700×700. The inversion of such a matrix would be a formidable task, even when utilizing computer techniques.

For this reason, iterative approximations to solutions have been developed by Scofield [2] and by Skarsgard, Johns, and Green [3]. An iterative technique similar to that described by the latter has been developed independently in this laboratory. Convergence criteria for this technique have been discussed by Geiringer [1]. In applying the technique, empirical evidence has been obtained for the validity of solutions obtained by this method. This evidence is discussed below. In addition, the propagation of error in the unfolding process is investigated in detail.

## 2. Iterative Solution

### 2.1. Procedure

Equation (1) may be written in matrix form as

P=RN.

Assume *N= U*_0_. Then this initial estimate will give

$$P_{0}=RU_{0}.$$

A measure of the closeness with which *U*_0_ represents the true *N* is given by the difference

$$\Delta_{0}=P-P_{0}.$$

*U*_0_ may be corrected to form

$$U_{1}=U_{0}+\Delta_{0},$$

and the new correction

$$\Delta_{1}=P-RU_{1}$$

is found. For the *n^th^* iteration

$$\begin{array}{l} U_{n}=U_{n-1}+\Delta_{n-1}\\ \Delta_{n}=P-RU_{n}\\ U_{n+1}=U_{r}+\Delta_{n} \end{array}\}.$$ (2)

It has been found for the present work that it is satisfactory to use *P* itself as the initial estimate *U*_0_. The technique has been used primarily in unfolding pulse height distributions obtained with the NBS Two Crystal Pair Spectrometer. Details of the detector and its response are described by Ziegler, Wyckoff, and Koch [4].

Various methods for arresting the iterative procedure may be used. In this work the data were unfolded using a predetermined number of iterations.

### 2.2. The Response Function Matrix

This section will discuss the problem of finding a matrix representation for the assumed analytic form of the response function. The response at pulse height *ϵ* due to one incident photon of energy *k*_0_ may be written [4]

$$\begin{array}{l} R\left(\epsilon ,k_{0}\right)\\ \;=\int_{0}^{k_{0}}dk\left[C_{1}\delta \left(k-k_{0}\right)+{C_{2}e-}^{\left(\frac{k_{0}-k}{C_{3}k_{0}}\right)}\right]\cdot C_{4}e^{-\frac{1}{2}{\left(\frac{k-\epsilon }{\alpha }\right)}^{2}},\\ \;\int_{0}^{k_{0}}R\left(\epsilon ,k_{0}\right)dk_{0}=1 \end{array}$$ (3)

where *C*_1_, *C*_2_, *C*_3_, *C*_4_, and *α* are constants characteristic of the detector. The pulse height distribution becomes

$$P\left(\epsilon \right)-\int_{0}^{\infty }dk_{0}R\left(\epsilon ,k_{0}\right)N\left(k_{0}\right)$$ (4)

where *N*(*k*_0_) is the continuous incident photon number spectrum. Equation (4) is the continuous form of (1). Experimentally the vector

$$P_{i}=\int_{\epsilon_{i}-\Delta_{i}}^{\epsilon_{i}+\Delta_{i}}P\left(\epsilon \right)d\epsilon$$

is the quantity measured as counts per channel in a multichannel pulse height analyzer.

The integral equation (4) does not possess an exact solution. Integrating over *k* in (3), (4) becomes

$$P(\epsilon )=\int dk_{0}\left\{C_{1}C_{4}\exp\left[-\frac{{\left(k_{0}-\epsilon \right)}^{2}}{2\alpha^{2}}\right]+C_{2}C_{4}\left(\alpha \sqrt{\frac{\pi }{2}}\right)\left[\exp\left(-\frac{1}{C_{3}}+\frac{\epsilon }{C_{3}k_{0}}+\frac{\alpha }{\sqrt{8}C_{3}^{2}k_{0}^{2}}\right)\right]\left[\Phi \left(\frac{k_{0}}{\sqrt{2\alpha }}-\frac{\epsilon }{\sqrt{2\alpha }}-\frac{\alpha }{\sqrt{2}C_{3}k_{0}}\right)-\Phi \left(\frac{\epsilon }{\sqrt{2}\alpha }+\frac{\alpha }{\sqrt{2}C_{3}k_{0}}\right)\right]\right\}N(k_{0})$$ (5)

where Φ(*x*) is the error integral [5]. Thus (5) is seen to be an integral equation with a Gaussian kernel; such an equation does not possess a general unique solution [6]. This is a manifestation of the inability to experimentally differentiate between a smooth spectrum and a spectrum containing a series of sharp spikes. The Gaussian broadening is responsible for this.

In order to obtain a matrix representation for *R*(*ϵ, k*_0_) a particular form must be assumed for *N*(*k*_0_). Two forms have been investigated. One may assume the incident spectrum to consist of a series of discrete steps so that over a fixed small energy width the spectrum is constant [7]. Alternatively one may assume the spectrum to be composed of a sum of Dirac delta functions so that when an integration is performed over a small energy width the area of the delta function gives the number of photons in that width.

Both cases lead to essentially the same form for the matrix. The latter case will be carried through to obtain the matrix explicitly.

Let $N\left(k_{0}\right)=\sum_{j}a_{j}\delta \left(k_{j}-k_{0}\right).$ Then (4) becomes

$$P(\epsilon )=\sum_{j}K_{j}f\left(k_{j},\epsilon \right)a_{j}$$ (6)

where $K_{j}=C_{1}C_{4}+k_{j}C_{2}C_{3}C_{4}\left(1-e^{-1/C_{3}}\right)$ is a number, and $f\left(k_{j},\epsilon \right)=e^{-\frac{1}{2}{\left(\frac{k_{i}-\epsilon }{\alpha }\right)}^{2}}$.

From the above the number of counts in channel *ϵ*_i_ is

$$P_{i}=\int_{\epsilon_{i}-\Delta_{i}}^{\epsilon_{i}+\Delta_{i}}\sum_{j}K_{j}f\left(k_{j},\epsilon \right)a_{j}$$

which, after interchanging integration and summation becomes

$$P_{i}=\sum_{j}a_{j}[K_{j}b\left(k_{j},\epsilon_{i},\Delta_{i}\right)]$$ (7)

where

$$b\left(k_{j},\epsilon_{i},\Delta_{i}\right)={\int_{\epsilon_{i}-\Delta_{i}}^{\epsilon_{i}+\Delta i}e^{-\frac{1}{2}\left(\frac{k_{i}-\epsilon }{\alpha }\right)}}^{2}d\epsilon =b_{ji}\left(\Delta_{i}\right).$$

Identifying *a_j_* with *N_j_* and *K_j_b_ij_* with *R_ij_* (7) becomes identical with (1).

## 3. Empirical Justification

### 3.1. Convergence

In setting out on this course there was no reason to believe the technique to be convergent. It has been shown [3] that convergence is assured for a smooth function if the eigenvalues Λ*_i_* of the response function matrix satisfy the requirement

$$0<\Lambda_{i}<2$$

This was not a useful test because the size of the matrices used made calculation of the eigenvalues impractical. Therefore the primary justification is empirical.

In analysis utilizing a 200 × 200 form of *R_ij_*, eleven iterations were ordinarily performed. However, as a check on convergence, as many as twenty-one iterations have been performed, during which Δ*_n_* of equation (2) continues to converge.

**Figure 1:**
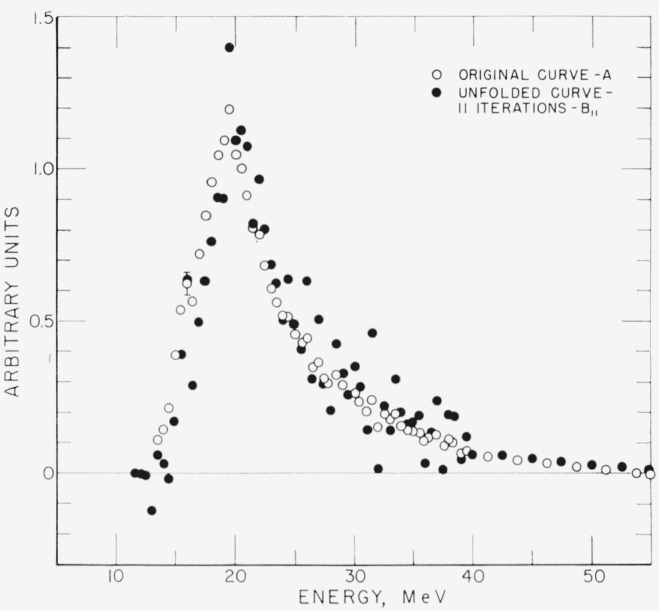
Original curve and the corresponding unfolded curve after eleven iterations, for a typical spectrum.

In figure 1 a typical set of points to be unfolded is plotted. Let *A* denote this set. On the same figure is plotted *B*, the result of unfolding *A.* The set *A* contained points only up to 40 MeV. In order to avoid introducing a large discontinuity in the first derivative at 40 MeV, a straight line tail has been added. The work was done with an energy grid width of 0.5 MeV. Typical standard deviation is shown for a point of *A* at 16 MeV.

**Figure 2:**
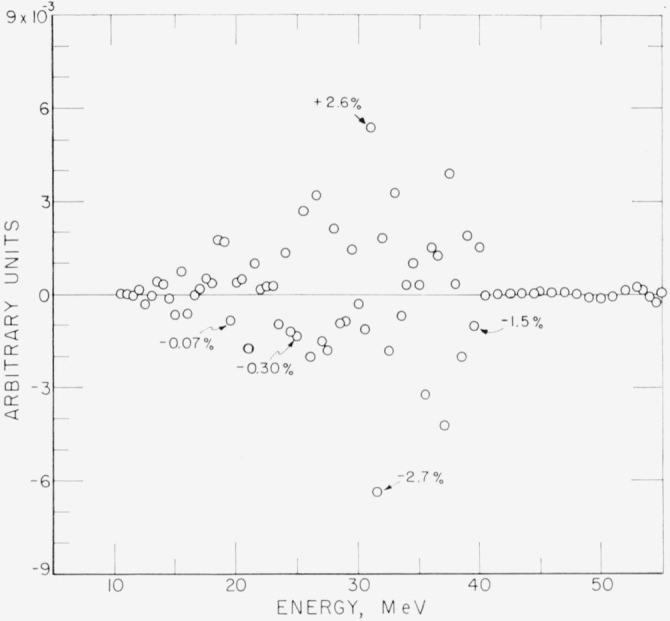
Difference, *Δ*, between the original curve of figure 1 and the folding of the unfolded curve of figure 1. X% indicates percentage difference Δ/A. Ordinate scale is in same units as figure 1.

In order to compare *B* with *A*, the difference Δ_11_*=A*−*RB* (see (2)) is plotted in figure 2. If *B* is the “correct” unfolded set of points then Δ_11_ must vanish. Convergence requires that Δ*_n_* vanishes for increasing *n.*

Some values of the difference in percent are indicated on the plot. The very small (0.7%) difference at 19.5 MeV is at the peak of *A.*

**Figure 3:**
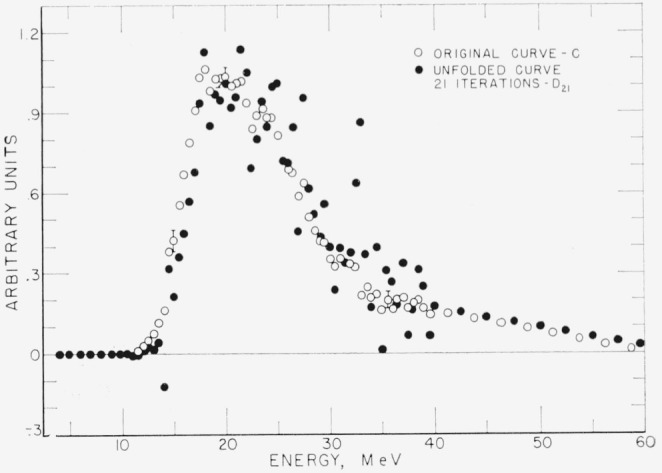
Original curve with poor statistics and unfolded curve after twenty-one iterations.

In order to further check the convergence properties of the scheme a set of points with large uncertainties was unfolded, using twenty-one iterations. The set *C* and its “unfold” *D* are shown in figure 3. Again a straight line tail has been added to *C* at 40 MeV.

Because of the poorer statistics on *C* there are more fluctuations in *D.* The slope of *C* appears to have a large discontinuity at 32.5 MeV. A spike in *D* is observed to grow at this energy with successive iterations. This demonstrates that fluctuations are magnified as one approaches an exact solution.

**Figure 4:**
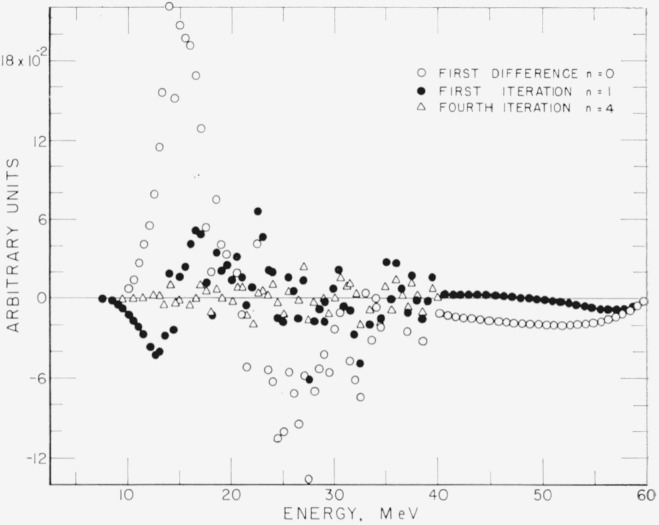
The difference Δ*_n_* between the original curve of figure 3 and the folding of the unfolded curve after *n* iterations, for n=0, 1, and 4. Ordinate scale in figures 4 and 5 is in the same units as figure 3.

**Figure 5:**
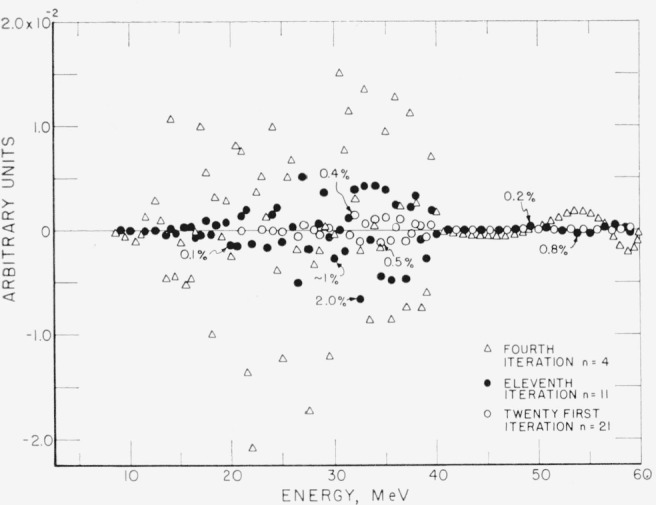
The difference Δ*_n_* for *n*=4, 11, and 21. Note that the ordinate scale is expanded from figure 4.

The question of convergence is best illustrated by examining Δ*_n_*, for various values of *n.* Figures 4 and 5 show Δ*_n_* for *n*=0,1,4,11, and 21. It is observed that Δ*_n_* converges rapidly for small *n.* The maximum of the ratio Δ_21_/Δ_0_ is approximately 10^−3^. The maxima of δ_21_ in percent of *C* are +0.4 and −0.5 percent.

A numerical criterion for testing convergence in this sense is suggested by Skarsgard, Johns, and Green [3] for a pulse height distribution containing pure Poisson counting errors, i.e. the standard deviation on *P_i_* is ${\sqrt{P}}_{i}$.

If $\frac{{\left(\Delta_{i}\right)}^{2}}{P_{i}}<<1$ then the deviation for the point *U_i_* is well within the limits of random measurement errors. Therefore if

$$\sum_{i=1}^{M}\frac{\Delta_{i}^{2}}{P_{i}}\leq M$$ (8)

the unfold is regarded as satisfactory. This test was used in unfolding a pulse height distribution for which the errors on each point were purely counting errors. The results were similar to those found by Skarsgard, Johns, and Green, [3] namely, convergence is rapid until (8) is satisfied (~3 iterations). After this convergence proceeds slowly.

One might hope to be able to prove convergence from the classical theorems [1]. It is easily shown that if one denotes (*I-R*) by *A*, then:

$$U^{\left(n\right)}-U_{r}=A^{n}\left(U_{0}-U_{T}\right).$$

Therefore [1] *U*^(^*^n^*^)^ converges to the true solution *U_T_* if and only if the eigenvalues of *A* are less than one in modulus. From the rapid convergence which is observed empirically, one is led to believe that the eigenvalues of *A* are indeed less than one in modulus.

A sufficient condition for convergence is that the maximum of the absolute row sums *μ_i_* satisfy

$$\mu_{i}=\left(\sum_{j=1}^{n}\left|A_{ij}\right|\right)<1.$$

However, this is not the case for the matrix *A=I-R* on which the present work is based.

## 4. Error Propagation

### 4.1. Empirical

**Figure 6:**
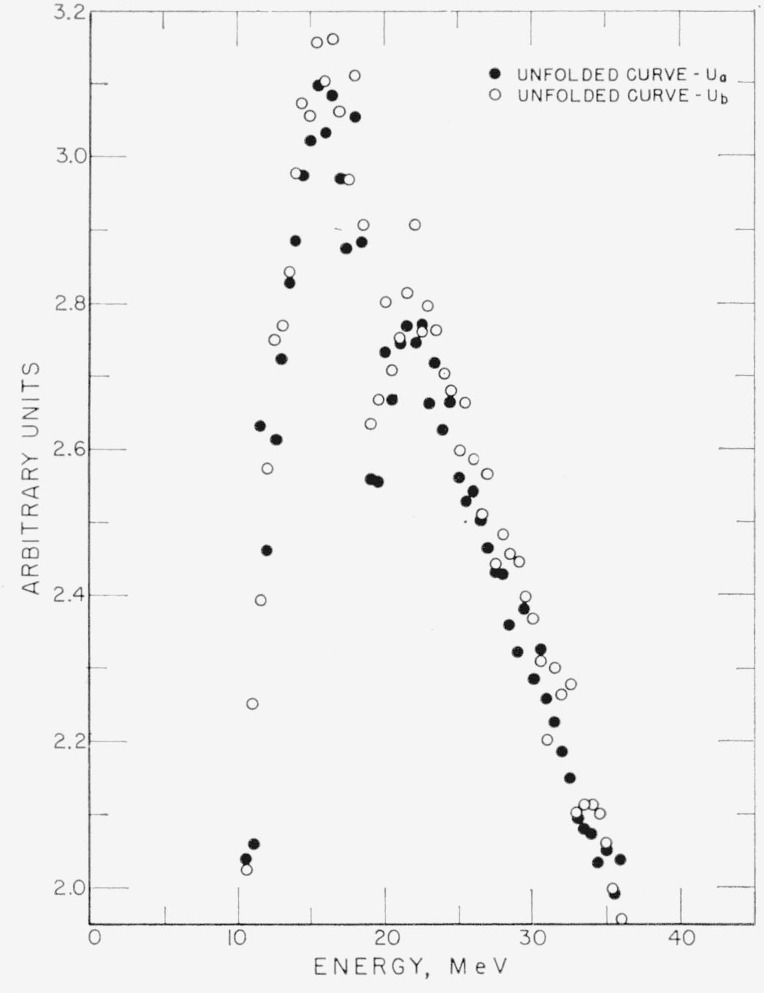
A portion of the unfolded spectra for two different experimental determinations of the same pulse height distribution after eleven iterations.

In order to demonstrate the effect of statistical fluctuations, two different experimental determinations of the same pulse height distribution have been unfolded. A portion of the unfolded spectra for both sets of data are presented in figure 6. The spectra are designated *U_a_* and *U_b_.* The two pulse height distributions are not presented because of the typographical difficulty in distinguishing the two sets of data on a meaningful scale.

**Figure 7:**
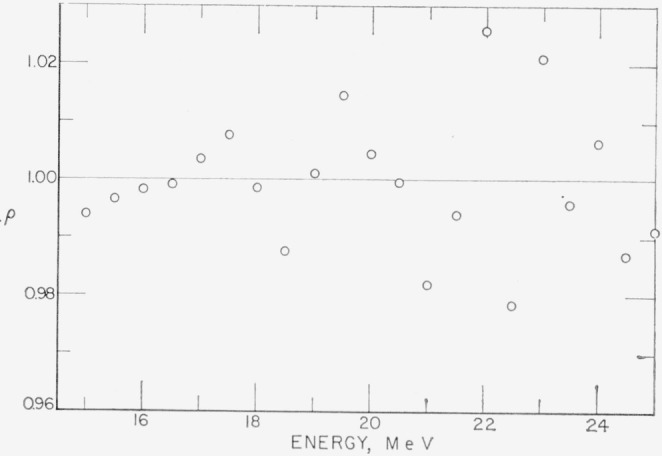
The ratio ρ=(*P_a_/U_b_*)/(*P_b_/U_b_*) See text for definitions.

Differences between the two spectra should be purely statistical. Let the measured pulse height distributions from which *U_a_* and *U_b_* were obtained be *P_a_* and *P_b_.* The ratio *ρ*=(*P_a_/U_a_*)*/*(*P_b_/U_b_*) has been plotted in figure 7 for the region from 15 MeV to 25 MeV. One would expect this ratio to be randomly distributed about unity due to the statistical fluctuations in *P_a_* and *P_b_.* This is observed.

In addition, if the unfolding procedure does not introduce false structure, then, for *P_a_>P_b_* one expects the relations *U_a_>U_b_* and therefore *ρ*>1, to hold approximately. Examination of figures 6 and 7 will show that for *U_a_*>*U_b_, ρ*>1, and for *U_a_<U_b_, ρ*<1, except when *U_a_*≃*U_b_* where fluctuations in adjacent points become important. This indicates that the iterative procedure does not introduce false structure.

Some qualitative effects of error propagation are illustrated quite well by figure 6. The most pronounced effect is the increase of fluctuations in the unfolded curves with increasing energies. The reason for this will emerge from the discussion following.

### 4.2. Calculation of Error

The calculation of error for an individual point in an unfolded spectrum is made difficult, because, in folding, correlations arise between errors in adjacent points.

Assume that error in the detected pulse height distribution is known as a function of energy and denote it by *σ_k_*. The folded set of points, which are obtained in the first step, may be written

$${\left[S\left(P\right)\right]}_{i}=\sum_{j}R_{ij}P_{j.}$$ (9)

The standard deviation of *S_i_* becomes

$$\delta_{i}=[\sum_{j}{\left(R_{ij}\sigma_{j}\right)}^{2}{]}^{\frac{1}{2}}.$$ (10)

From (2) the solution after one iteration may be written

$$U_{i}^{\left(1\right)}=2P_{i}-\sum_{j}R_{ij}P_{j}$$ (11)

with corresponding error

$$\delta U^{\left(1\right)}{\;}_{i}=[{\left(2-R_{ii}\right)}^{2}{\sigma_{i}}^{2}+\sum_{j\neq i}{\left(R_{ij}\sigma_{j}\right)}^{2}{]}^{\frac{1}{2}}.$$ (12)

The second iteration gives:

$$\begin{array}{c} U^{\left(2\right)}=U^{\left(1\right)}+P-2RP+R^{2}P\\ =3P-3RP+R^{2}P \end{array}$$

where

$$R^{2}P=\sum_{jk}R_{ij}R_{jk}P_{k}.$$

Expanding *U*^(^*^2^*^)^ in the same way as *U*^(^*^1^*^)^, the error on *U*^(^*^2^*^)^ becomes

$$\delta U_{i}{\;}^{\left(2\right)}={\left[\left(3-3R_{ii}+R_{ii}{\;}^{2}\right){\sigma_{i}}^{2}+\;\text{other}\;\text{terms}\right]}^{\frac{1}{2}}.$$

The “other terms” have numerous cross products. For example, the contribution to ${\left[\delta U_{i}^{\left(2\right)}\right]}^{2}$ from *σ_i_*_+1_ is [−3*R_i,__i_*_+1_+*R_ii_R_i,__i_*_+1_+*R_i_*, *_i_*_+1_, *R_i_*_+1, i+1_]^2^*σ_i_*_+1_^2^.

The general form for the solution after *n* iterations may be written symbolically as

$$U^{\left(n\right)}=\frac{1}{R}\left[I-{\left(I-R\right)}^{n+1}\right]P$$ (13)

where is the identity matrix and *1/R=R*^−^^1^. If the variance Var (*P*) *= σ*^2^*I*, then the variance of *U*^(^*^n^*^)^ may be written formally as:

$$\text{Var}\left(U^{\left(n\right)}\right)=R^{-1}\left[I-{\left(I-R\right)}^{n+1}\right]\;{\left[I-{\left(I-R\right)}^{n+1}\right]}^{T}{\left(R^{-1}\right)}^{T}\sigma^{2},$$

where *T* denotes the transpose. Here we have used [8]

$$\text{Var}\left(CP\right)=C\left[\mathrm{var}\left(P\right)\right]C^{T}.$$

Since all elements of *R* are less than unity, it is evident that in the limit as *n* approaches infinity

$$\underset{n\to \infty }{\lim}\text{Var}\left(U^{\left(n\right)}\right)=R^{-1}{\left(R^{-1}\right)}^{T}\sigma^{2}.$$

**Figure 8:**
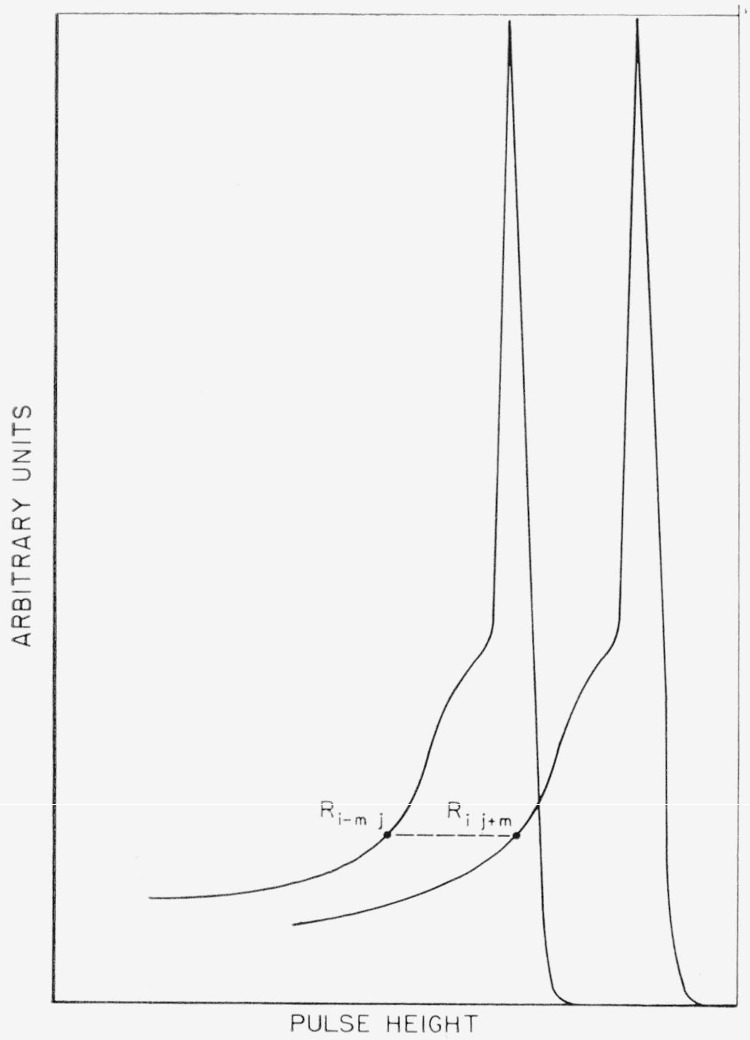
Illustration of the relation between the response functions at two different energies. The folded spectrum at *i* is *R_ii_P_i_+R_i i_*_+1_*P_i+_*_1_+… where *R* and *P* are defined in the text. *R_i i+_*_1_≃*R_i_*_−1, i_.

In order to simplify the error calculation the response function and the error will be assumed to satisfy the following conditions:

1. The half-width of the response function is narrow. This corresponds to good resolution in the detector.
2. The shape of the response function does not change rapidly with incident photon energy. This is equivalent to assuming that
  $$\begin{array}{c} R\left(\epsilon ,k_{1}\right)≃R\left(\epsilon +\Delta k_{1},k_{1}+\Delta k_{1}\right)\\ \text{or}\;R_{ij}≃R_{i+m,j+m\;}\;\text{and}\;R_{i-m,j}≃R_{i,j+m,} \end{array}$$
  where *m* is an integer. See figure 8.
3. The error, *σ_k_*, is a constant, *σ*, over the half-width of the response function. Note that the first condition makes this more likely.

Let *R_ii_=w*_0_, *R_i−_*_1_,*_i_=w*_1,_ *R_i_*_−2_,*_i_=w*_2_, … But, from condition (2) *R_i_,_i+k_*≃w*_k_.* Using this (9) may now be written

$${\left[RP\right]}_{i}=\sum_{k}w_{k}P_{k+i},$$ (9a)

where the *w_k_* may be obtained from the response at one incident energy.

In the appendix it is shown that the solution has the general form:

$$U_{i}^{\left(n\right)}=a_{0}P_{i}+a_{i}P_{i+1}+a_{2}P_{i+2}+\ldots$$ (14)

If the error is assumed to be a constant, *σ*, this gives

$$\delta U_{i}{\;}^{\left(n\right)}={\left(\sum_{j}a_{j}^{2}\right)}^{\frac{1}{2}}\sigma$$ (15)

for the error at point *i* in the *n*th iterated solution.

For the response described in [4] the error after three iterations has been calculated at 18.5 MeV and 48.5 MeV. (See appendix.) The results are ±2.36 *σ* at 18.5 MeV and ±3.23 *σ* at 48.5 MeV.

In both cases if all terms in (*A*3) which are cubic in *w* were omitted, the difference in *U_i_*^(3)^ would be small, and the difference in *δU_i_*^(3)^ would be negligible. The terms which are cubic in *w* are approximately an order of magnitude smaller than the quadratic terms. The conclusion is that *δU*^(*n*)^ has converged for n≥3.

From (*A*1), (*A*2), and (*A*3) it may be observed that after a large number of iterations the coefficient of *P*_o_ in (*A*3) will converge to:

$$a_{0}=\left[1-{\left(1-w_{0}\right)}^{n+1}\right]/w_{0}.$$

For the cases at 18.5 and 48.5 MeV this gives *a*_o_=2.21 and 3.104 respectively, for *n*=3. Comparison with the results for *δU_i_*^(3)^ above shows very close agreement. Thus one concludes that three iterations satisfy the large number criterion.

**Figure 9:**
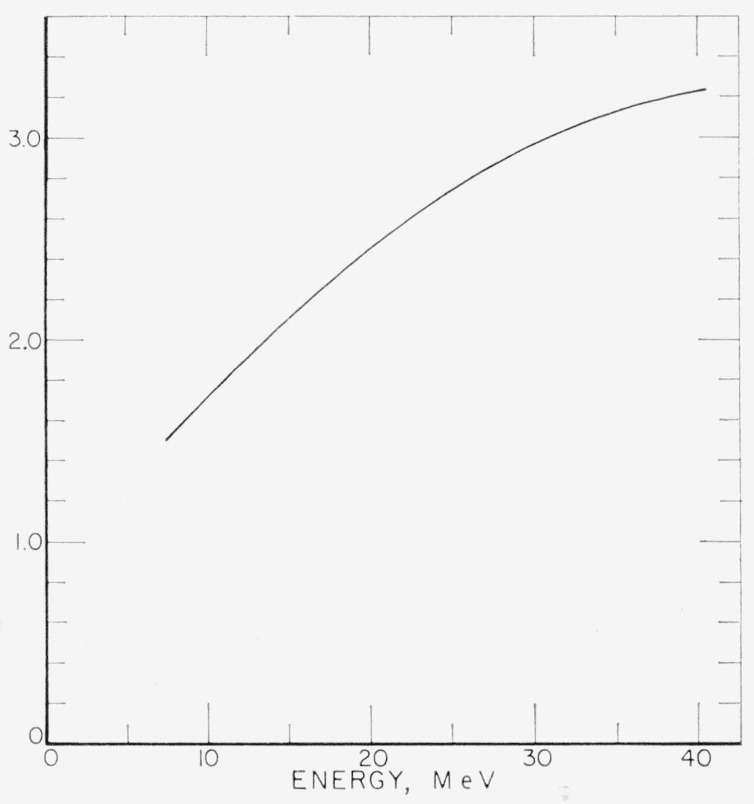
The function *a*_0_ = [1 − (1−*w*_0_)^*n*+1^]/*w*_0_ as a function of energy for *n*=3.

This coefficient then places a lower limit on the propagated error at each point of the solution. In general *w*_o_ decreases with increasing incident photon energy, for this experiment. Therefore the error must increase with energy. This is independent of the shape of the curve to be unfolded. Figure 9 shows *a*_o_ plotted as a function of energy for *n*=3.

## 5. Summary

Because of the uncertainties associated with any point on a measured pulse height distribution, any “solution” for the unfolded spectrum is acceptable, if the difference between the measured distribution and the fold of the solution lies within the uncertainty associated with the measured distribution. The additional requirement of smoothness is sufficient to ensure that the iterative process converges to a useful solution.

General error analysis is difficult. However, approximations may be made which become better as the resolution of the detector improves; these approximations make an error estimate possible.

Interesting results from the use of this technique may be seen in the work of Ziegler, Koch, Wyckoff, and Uhlig [9].

## 6. Appendix

In this appendix it will be shown how the general form of the solution, equation (14), may be found.

Using (9a) and expanding (13) the general solution becomes:

$$U_{t}^{\left(n\right)}=nP_{i}-\frac{n\left(n-1\right)}{2!}\sum_{j}w_{j}P_{j+i}+\frac{n\left(n-1\right)\left(n-2\right)}{3!}\sum_{k}\sum_{j}w_{k}w_{j}P_{k+j+i}+\frac{n\left(n-1\right)\left(n-2\right)\left(n-3\right)}{4!}\sum_{l}\sum_{k}\sum_{j}w_{l}w_{k}w_{j}P_{l+k+j+i}+\ldots$$ (Al)

Choosing an arbitrary zero point for index *i* (Al) may be written:

$$U_{0}^{\left(3\right)}=4P_{0}-6\sum_{j}w_{j}P_{j}+4\sum_{j}w_{j}^{2}P_{2j}+8\sum_{k}\sum_{<j}w_{k}w_{j}P_{k+j}-\sum_{j}w_{j}^{3}P_{3j}-3\sum_{k}\sum_{<j}w_{k}^{2}w_{j}P_{2k+}-3\sum_{j}\sum_{<k}w_{k}^{2}w_{j}P_{2k+j}-6\sum_{j}\sum_{<k}\sum_{<j}P_{l+k+j.}$$ (A2)

Expanding and collecting terms:

$$\begin{array}{l} U_{0}^{\left(3\right)}=(4-6w_{0}+4w_{0}^{2}-w_{0}^{3})P_{0}+(-6w_{1}+8w_{0}w_{1}-3w_{0}^{3}w_{1})P_{1}\\ \;+(-6w_{2}+4w_{1}^{2}+8w_{0}w_{2}-3w_{0}^{2}w_{2}-3w_{0}w_{1}^{2})P_{2}\\ \;+(-6w_{3}+8w_{0}w_{3}+8w_{1}w_{2}-w_{1}^{3}-3w_{0}^{2}w_{3}-6w_{0}w_{1}w_{2})P_{3}+(-6w_{4}+4w_{2}+8w_{0}w_{4}+8w_{1}w_{3}-3w_{0}^{2}w_{4}-3w_{1}^{2}w_{2}-3w_{2}^{2}w_{0}-6w_{0}w_{1}w_{3})\\ \;P_{4}+(-6w_{5}+8w_{0}w_{5}+8w_{1}w_{4}+8w_{2}w_{3}-3w_{0}^{2}w_{5}-3w_{1}^{2}w_{3}-3w_{2}^{2}w_{1}-6w_{0}w_{1}w_{4}-6w_{0}w_{2}w_{3})P_{5}+\ldots \end{array}$$ (A3)

At 18.5 MeV the response function for the detection system described by Ziegler, Wyckoff, and Koch [4] was given by

$$\left(w_{0},w_{1},w_{2},w_{3},w_{4},w_{5},\ldots \right)=\left(0.39,0.225,0.138,0.088,0.056,0.035,\ldots \right).$$

Using these values in (A3) one finds:

$$U_{0}^{\left(3\right)}=2.209P_{0}-0.751P_{1}-0.317P_{2}-0.129P_{3}-0.084P_{4}-0.003P_{5}-\ldots ,$$

and from (15):

$$\delta U_{0}{\;}^{\left(3\right)}=2.36\sigma .$$

Similarly the response at 48.5 MeV gives

$$U_{0}{\;}^{\left(3\right)}=3.103P_{0}-0.642P_{1}-0.454P_{2}-0.333P_{3}-0.242P_{4}-0.174P_{5}-\ldots ,$$

which leads to

$$\delta U_{0}{\;}^{\left(3\right)}=3.23\sigma .$$
